# Evaluating the link between predation and pest control services in the mite world

**DOI:** 10.1002/ece3.6655

**Published:** 2020-08-15

**Authors:** Lise Roy, Adrien Taudière, Julien Papaïx, Rumsais Blatrix, Geoffrey Chiron, Ghais Zriki, Olivier Bonato, Jean‐Yves Barnagaud

**Affiliations:** ^1^ CEFE University of Montpellier CNRS EPHE, IRD Université Paul‐Valéry Montpellier 3 Montpellier France; ^2^ UR 546 BioSP INRA Avignon France; ^3^ Institut Technique de l'AVIculture (ITAVI) Lyon Lyon Cedex 07 France; ^4^ Interactions Plantes Microorganismes Environnement (IPME) IRD Cirad, UM Montpellier France; ^5^ CEFE University of Montpellier CNRS EPHE‐PSL University IRD Université Paul‐Valéry Montpellier 3 Montpellier France

**Keywords:** animal farming, assemblage dynamics, biological control, ecosystem services, predation, trophic interactions

## Abstract

1. Pest regulation by natural enemies has a strong potential to reduce the use of synthetic pesticides in agroecosystems. However, the effective role of predation as an ecosystem service remains largely speculative, especially with minute organisms such as mites.
2. Predatory mites are natural enemies for ectoparasites in livestock farms. We tested for an ecosystem level control of the poultry pest *Dermanyssus gallinae* by other mites naturally present in manure in poultry farms and investigated differences among farming practices (conventional, free‐range, and organic).
3. We used a multiscale approach involving (a) in vitro behavioral predation experiments, (b) arthropod inventories in henhouses with airborne DNA, and (c) a statistical model of covariations in mite abundances comparing farming practices.
4. Behavioral experiments revealed that three mites are prone to feed on *D. gallinae*. Accordingly, we observed covariations between the pest and these three taxa only, in airborne DNA at the henhouse level, and in mites sampled from manure. In most situations, covariations in abundances were high in magnitude and their sign was positive.
5. Predation on a pest happens naturally in livestock farms due to predatory mites. However, the complex dynamics of mite trophic network prevents the emergence of a consistent assemblage‐level signal of predation. Based on these results, we suggest perspectives for mite‐based pest control and warn against any possible disruption of ignored services through the application of veterinary drugs or pesticides.

## Graphical abstract

In vitro tests have been confronted with mite inventories and airborne DNA analyses. Three out of seven taxa of native predatory mites are able to feed on *Dermanyssus gallinae*. The same three predators are associated with the parasite in poultry houses. We've detected no obvious signals from any pest control service. This may be due to the ubiquity of predators or to prey–predator dynamics.

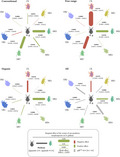

## 1 INTRODUCTION

Populations and assemblages in natural ecosystems are shaped by the interaction of bottom‐up forces (resource limitation) and top‐down forces (consumer regulation) (Leroux & Loreau, 2015). In agroecosystems, the bottom‐up forces centered on the organism under production are fixed at a high level by human inputs (fertilizer, animal feed). Nevertheless, many top‐down and bottom‐up forces involve uncultivated organisms that interact at various levels. The setting of a biological control requires that antagonistic interactions, such as predation, trigger a top‐down trophic cascade sufficient to regulate a pest in nonlimiting resource conditions (as defined by Ripple et al., 2016). If efficient at the ecosystem level, biological pest control can contribute significantly to reduce the use of agrochemicals, and particularly insecticides. The emergence of such process is often hypothesized to be favored by high biodiversity, assumed to multiply interacting top‐down and bottom‐up forces as a consequence of the complexification of trophic networks. The association between biodiversity and yields in agricultural landscapes varies, however, from highly positive to nil or even negative (Karp et al., 2018). Uncovering the relationships among regulating processes and species dynamics within assemblages is therefore necessary to evaluate the existence of an ecosystem service.

The link between a predation process and the associated service (pest control) is difficult to quantify at the scales of farms and agricultural landscapes, because it rarely emerges additively due to assemblage‐level processes. The coexistence of multiple predators that attack a given pest may, as a consequence, either heighten or downplay the benefits of biological control (Caballero‐López et al., 2012; Colfer & Rosenheim, 2001; Janssen et al., 2006; Losey & Denno, 1998). In addition, density‐dependent interactions between the prey and its own resources can promote positive effects of predation (Abrams, 1992). Furthermore, pest–predator interactions and the resulting control service at an ecosystem level are strongly modulated by landscape composition and configuration (e.g., landscape complexity in Perez‐Alvarez, Nault, & Poveda, 2019, and Winqvist et al., 2011, forest cover in Librán‐Embid, De Coster, & Metzger, 2017). These effects typically range from positive to negative within single agroecosystems, with no obvious rule. The idiosyncratic nature of species responses to environmental variation, the complexity of biotic interactions, and the multidimensional multiscale structure of species assemblage may therefore hide clear associations between a given process and higher‐level pattern (Micheli et al., 1999).

The link between predation and pest control services is generally assumed more than actually tested because both predation itself and the associated service are difficult to measure, especially when dealing with arthropod pests (Kremen & Miles, 2012). Most studies characterize either predation capacity in vitro with arthropods considered promising for biological control (e.g., Knoll, Ellenbroek, Romeis, & Collatz, 2017), or quantify the effects of various factors (landscape complexity, agricultural practices, etc.) on the diversity of predator guilds in agroecosystems (e.g., Flohre et al., 2011; Hedde et al., 2015; Paoletti, Schweigl, & Favretto, 1995; Weibull & Östman, 2003). Only a handful of studies have combined methods from community ecology with tedious field experiments to evaluate the link between predation and a pest control service. For example, Perez‐Alvarez et al. (2019) and Winqvist et al. (2011) measured the relationships between the diversity of predator guilds and the frequency of predation events recorded on artificially exposed pest individuals in the field. Others seek to determine whether the damage recorded on crops is significantly higher in modalities that exclude predators (e.g., nets impassable to birds and bats but permeable to butterfly pests Librán‐Embid et al., 2017). These rare integrative studies have all revealed contrasting effects within a given ecosystem, that would have been missed by simpler studies. Painstaking explorations at various levels are thus needed in order to understand the full chain of processes involved in a pest control service, but may not be conclusive due to this complexity.

The type of farming practice can influence the expression of a given ecosystem service. Certified organic agriculture aims at ecological intensification, in particular by prohibiting the use of synthetic insecticides. Although the effect of landscape often surpasses that of practices applied within agricultural plots (Bengtsson, Ahnström, & Weibull, 2005; Weibull & Östman, 2003; Winqvist et al., 2011), Muneret et al. (2018) show that organic farming practices overall do favor the processes leading to services. However, this meta‐analysis also detects a higher rate of pest infestation in organic farms than in nonorganic ones. Interestingly, the detection of the positive effect of organic practices is highly dependent on the indicator used: higher detection using functional traits of predatory insects than their taxonomic diversity (Hedde et al., 2015), for instance. Furthermore, pest species richness, not abundance or dominance, modulates the efficiency of pest control service (Dainese et al., 2019). The dependence to the choice of indicators suggests that exploring the emergent properties of pest–predator assemblages is a good way to advance the understanding on how agricultural practices affect the pest control service.

Hematophagous arthropods such as midges, bedbugs or poultry mites cause significant damage in animal production, but few studies addressed their regulation by natural enemies. These hematophagous arthropods are naturally prone to encounter various predators within livestock buildings as they spend most of their lives at a distance from their hosts in microhabitats such as cracks and crevices. Many predatory arthropods that do not interact directly with vertebrate hosts colonize livestock buildings from the surrounding environment by way of phoresis on manure‐dwelling insects (e.g., in poultry, Brady, 1970a, 1970b; Horn, Körbes, Granich, Senter, & Ferla, 2016; Roy et al., 2017). The only published attempt to use native arthropods for biological control at the scale of a livestock production farm, to our knowledge, aimed to regulate nonhematophagous coprophilous flies in poultry production through amendments of the manure management (Hinton & Moon, 2003). However, whether predation affects hematophagous populations at the scale of poultry farms arthropod assemblages remains unknown, nor whether organic practices promote these processes. The development of biological control in animal husbandry generally lags far behind that of crop production, in spite of being a foreground challenge in the current context of society's demand for a compromise between food safety and environmental protection, including pesticide reduction and animal welfare. Furthermore, land applications of recycled livestock manures are increasing worldwide (Motoyama et al., 2011) and generate environmental pollution by drug residues (Kaczala & Blum, 2016). Reducing such treatments in livestock housing is expected to reduce toxic residues in food of animal origin and substantially mitigate the impact of pesticides on biodiversity in farmlands at once.

The poultry red mite *Dermanyssus gallinae* (De Geer, 1778) is a hematophagous mite that impairs poultry health and welfare, as a reservoir of *Salmonella* spp. (Valiente Moro, Chauve, & Zenner, 2007). Its widespread occurrence results into substantial economic impacts on the egg‐producing sector worldwide (Sparagano, George, Harrington, & Giangaspero, 2014; Valiente Moro et al., 2007). The control of this pest mainly relies on synthetic neurotoxic acaricides (Brauneis, Zoller, Williams, Zschiesche, & Heckeroth, 2017; Chauve, 1998), which increases environmental pollution risk through contaminated manures. Implementing biological control against *D. gallinae* is in line with the current challenges defined by the One‐Health initiative, a transdisciplinary approach to health management integrating human and veterinary medicine with environmental sciences (Destoumieux‐Garzón et al., 2018; Lerner & Berg, 2017). Lab‐reared predatory mites have been sold in recent years for inundative biological control, although with limited success (Knapp, van Houten, van Baal, & Groot, 2018). Native mite assemblages from British, Brazilian, and French layer farms have been shown to be relatively rich (Brady, 1970a, 1970b; Horn, Granich, Horn Körbes, Liberato Da Silva, & Ferla, 2018; Roy et al., 2017), and several taxa have been shown to feed on *D. gallinae* in vitro (Zriki, Blatrix, & Roy, 2020). Nevertheless, no study has, to date, attempted to assess whether naturally occurring predators could affect the dynamics of *D*. gallinae.

Interactions between mites are difficult to test in their environment (Lindquist, 1975). They are small, often highly mobile, and live mainly in coarse substrates (manure, dust, soil) with grain sizes similar to those of prey and predators, impairing visual detection. Many studies have focused on predation among mites in highly simplified systems (rarely more than two species within in vitro systems; see Solomon, 1969, for review, Janssen, van Gool, Lingeman, Jacas, & van de Klashorst, 1997). Field demonstrations of control services by predatory mites in livestock farms are severely hampered by the low specificity of aggression marks on hosts (no typical lesions can be observed on birds as opposed to those produced on plants by crop pests), and in situ experiments are practically unfeasible. Even more generally, the effect of predation at the scale of mite assemblages has not really been tested and its consequences for pest regulation services not evaluated.

In the present study, we provide the first attempt to investigate mite predation on *D. gallinae* in poultry farms from the individual to the assemblage levels. We tested predation on *D. gallinae* through in vitro tests on mites taken directly from the field. Complementarily, we tested for a signal of predation at the level of mite assemblages through a statistical comparison of prey–predator correlations in three farm types (conventional, free‐range, and organic), using metabarcoding on airborne DNA and a rapid biodiversity assessment method on manure mites.

We specifically tested the following three hypothesis‐question pairs:

- naturally occurring mites can feed on the species of interest: Is predation on *D. gallinae* by naturally occurring mites possible?
- the prey and its predators are present together in the same henhouse, a prerequisite for biological pest control to take place: Can predation take place in real life?
- farming practices interfere with this possible service, with organic farms probably being the most favorable: Does this predation differ from one farming practice to another?

## 2 MATERIAL AND METHODS

### 2.1 System under study and sampling strategy

#### 2.1.1 Mites under test

We tested the interactions between the target pest *D. gallinae* and seven of the mite morphospecies defined in Roy et al. (2017), which consist in either a single or multiple true species, although all are taxonomically distinct (see Table 1). These mites were chosen among those most frequently recorded in henhouse manures and possible predators of mites according to literature.

**TABLE 1:** Morphospecies considered. These distinct taxonomic entities, characterized by Roy et al. (2017), are distinguishable alive with a stereomicroscope, without recourse to their microscopic preparation

Morphospecies ID	Taxonomic content
Dg	*Dermanyssus gallinae* (Dermanyssoidea)
CHE	*Cheyletus* spp. (Cheyletoidea)
ME1	*Dendrolaelaps presepum* (dominant species), *Dendrolaelaps* sp., *Halolaelaps* sp. (Rhodacaroidea)
ME2	*Androlaelaps casalis* (incl. two cryptic species; Dermanyssoidea)
ME4	*Proctolaelaps parascolyti* (Ascoidea)
ME7	*Macrocheles muscaedomesticae* (dominant species), *Macrocheles* spp. (Eviphidoidea)
UR1	*Uroobovella fimicola* (Uropodina)
UR2	*Trichouropoda orbicularis, Uroobovella marginata, Nenteria* sp. (Uropodina)

**FIGURE 1:**
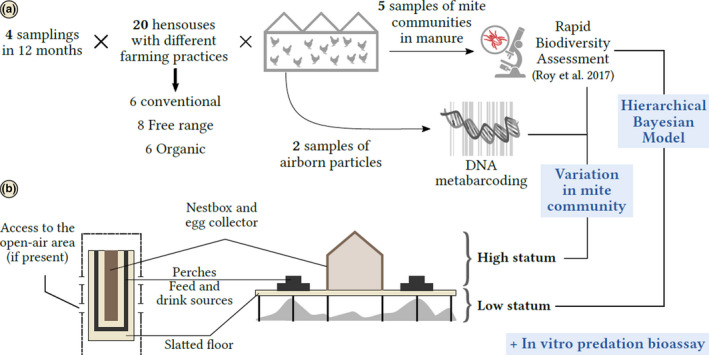
Sampling information. (a) Sampling design: 20 barn henhouses (in 16 farms) located in two different landscapes in Southeastern France (Ain and extended Drôme). Four successive sampling campaigns were processed, with effective airborne particles and manure sampling in each of farm buildings hosting a layer flock at the moment of sampling campaign. (b) Typical layout of a barn henhouse. *Right,* internal vertical cross‐sectional view of a henhouse. *Left,* top view of a barn henhouse with an open‐air area (free‐range and organic types). In conventional farms, the trapdoors are just kept closed.

In farms, the prey *D. gallinae* remains strictly confined to the henhouses. Inside, it tends to reside preferentially in the upper stratum (where the hens are), while some predators (mesostigmatid mites) are often concentrated in the lower stratum (in manure under the slatted floor, Figure 1) and some others (*Cheyletus* spp.) are more evenly distributed between the two strata. However, even the predators that concentrate on the lower stratum are expected to impact the prey since they are vagile and active hunters that often travel between the low and high strata (Maurer, Baumgärtner, Bieri, & Fölsch, 1993; LR pers. obs.).

#### 2.1.2 Study sites

The study involved 6 conventional, 8 free‐range, and 6 organic barn henhouses (Figure 1a) located in eastern France down to Jura mounts (Ain region; 10 henhouses; 1 organic, 5 conventional, 4 free‐range) and south to the Rhône valley (Drôme region; 10 henhouses; 5 organic, 2 conventional, 3 free‐range). The three farm types primarily differed in hen density (lowest in organic farms), pesticide and drug use (synthetic in free‐range and conventional versus of natural origin in organic farms), and whether hens can accede an outdoor space (absent in conventional only, see Appendix S1).

The layout of French barn henhouses is similar in the three farm types however (Figure 1b). The inside always contains the basic equipment necessary for the hens. It is placed on the slatted floor (high stratum, Figure 1b) under which manure accumulates (low stratum).

#### 2.1.3 Sampling design and mite inventories

We conducted four successive sampling campaigns in 2016 at 3‐month intervals: March (13–22nd), June (9–15th), September (19–22nd), and December (12‐15th). During each campaign, we systematically sampled manure and airborne particles from each henhouse (see below). Henhouses not operating at the time of a given campaign (empty period for sanitation once a year) were excluded from this campaign, and one henhouse was not sampled in September due to sanitary impediments. The start dates of the flocks (introduction of a new group of producing hens into a henhouse after a sanitary empty period) varied among farms; thus, the age of the flock varied between the farms at each sampling campaign (hereafter “flock age”). Additional campaigns were conducted to sample live predators for in vitro tests (see below).

During each sampling campaign and in each henhouse, we sampled airborne particles from two randomly selected points from ca. 30 cm above the slatted floor using a Coriolis^®^ µ air sampler (Bertin Instruments). Airborne particles were collected into a PBS + 0.01% Tween32 medium at a rate of 0.1 m^3^ per min for 10 min. A 100–110 bp long DNA fragment of the variable region V7 in the gene coding the 18S rRNA from all Eukaryotes was amplified by PCR using the following primer pair: forward: 5′‐TTTGTCTGSTTAATTSCG‐3′ and reverse 5′‐CACAGACCTGTTATTGC‐3′ (Guardiola, et al., 2015). The PCR products were sequenced via Illumina MiSeq by Spygen. The obtained sequences were analyzed using the bioinformatics pipeline described in Appendix S2. In short, sequences were quality‐filtered using Sickle (https://github.com/najoshi/sickle) and clustered into operational taxonomic units (OTUs) using vsearch (Rognes, Flouri, Nichols, Quince, & Mahé , 2016). Finally, each OTU was taxonomically classified using RDP‐classifier (Wang, Garrity, Tiedje, & Cole, 2007).

We also collected five standard manure samples from randomly selected points, during each sampling campaign and in each henhouse (*n* = 20 x 4 x 5), to characterize manure mite assemblages following the methodology in Roy et al. (2017). Each sample consisted of 250 ml of manure taken from an area approximately 40 cm in diameter and 5 cm deep in a glass flask sealed with a rubber stopper. Exposure to saturating ethyl acetate vapors within one hour of sampling killed the arthropods and prevented further development of the assemblage.

All arthropods from these samples were identified and counted using the Rapid Biodiversity Assessment method developed by Roy et al. (2017) for sorting out mite morphospecies and counting them using a binocular stereomicroscope.

### 2.2 Is predation on *D. gallinae* by naturally occurring mites possible?

We used in vitro tests to estimate the voracity of mites and their preference for *D. gallinae* over other farm‐dwelling preys. We also assessed whether alternative mites may divert predators from the focus prey and differentiated predation among stages of *D. gallinae*.

We conducted tests by exposing pairs of mite preys for 24 hr to single starved predators individualized in transparent wells in microplates (El Adouzi et al., 2017). One test consisted of a well containing one putative predatory mite and a protonymph of *D. gallinae* plus either an Astigmatid mite (an alternative prey species commonly occurring in poultry farms) or an adult female *D. gallinae*. Test modalities were defined by the putative predator species and the identity of the alternative prey (adult female *D. gallinae* or Astigmata) accompanying the protonymph of *D. gallinae*. Several different modalities were tested on the same microplate and at the same time (= a series), with at least 15 test replicates (i.e., 15 mite predators in 15 microplate wells) per modality. To minimize the effect of confounding factors, (a) we tested each modality on different microplates successively (min. two), (b) we tested several modalities together in each series, and (c) we randomly rearranged the set of modalities tested together in each series. For each modality in each series, we tested a similar number of prey pairs in the absence of predator as controls to estimate their natural mortality during test.

We tested two commercially available species of Mesostigmatid mites (*Stratiolaelaps scimitus* and *Macrocheles robustulus)* as controls to validate the in vitro test system. These two mites are readily available in large numbers, and both are generalist soil mite predators. We thus checked that mite predators were able to frequently feed on *D. gallinae* under our design. Live native predatory mites used in tests were collected from some of the henhouses sampled during the study.

Distinguishing between natural death of a prey and death due to predation is often impossible since mite predators mainly ingest liquids and do not swallow the prey body (Koehler, 1999). Therefore, we calculated a corrected prey mortality following Abbott (1925) to account for natural mortality of preys as estimated in controls.

We estimated the specific prey preferences for each predator using two‐tailed binomial tests based on the null hypothesis that the probability of scores for one or the other prey is equal to 50% (the level of significance was 5% with Bonferroni correction). In these analyses, we considered only predators that killed a single prey, assuming that the others had made no choice.

### 2.3 Can predation take place in real life?

Air DNA data provide a reasonable sampling of the mite assemblage composition throughout a henhouse as permanent ventilation shuffles the low and high strata permanently. We assigned each mite OTU from air DNA metabarcoding to one of the studied morphospecies to characterize mite assemblages from the entire henhouse. To do so, we compared OTU sequences with sequences previously obtained using classic Sanger sequencing from manure sampled in a previous study on the same set of farms (Roy et al., 2017).

We computed Spearman's rank correlations between the relative proportions of predatory morphospecies and *D. gallinae* in air DNA in search of a covariation between manure‐dwelling predators and the pest at the whole‐henhouse scale. We used the relative proportion instead of absolute values because the relationship between the number of sequences and the number of individuals varies sharply between taxa.

We compared the occurrence of each morphospecies in air (DNA) and manure (mite counts) data to assess the quality of the air data. We used Spearman's coefficients to correlate counts of morphospecies averaged from five manure samples per henhouse against counts of corresponding OTUs from two air samples per henhouse to refine the comparison.

### 2.4 Does this predation differ from one farming practice to another?

We related the covariations in manure counts of *D. gallinae* to those of its putative predators in a hierarchical multispecies model implemented in a Bayesian framework (see details in Appendix S3). This additional analysis permitted to assess whether farm type, region, season, and flock age modulated a possible signal of predation at the scale of mite assemblages within henhouses. We decided to focus on manure data for this analysis so as to work on absolute values (direct mite inventories) rather than ratios of environmental DNA (proportion of sequences of the different mite species). In short, this model described count variations of *D. gallinae* and its predators as Poisson distributions. We used mite counts as covariates explaining variations in *D. gallinae* counts, in interaction with four other covariates: farm type (conventional, free‐range, organic), season (four seasons), flock age (the age of hens at the moment of sampling), and region (two regions). Farm types differed by hen density, nature of chemical inputs, and access to an outdoor space. Thus, the respective effects of these factors could not be disentangled and we used farm type as an integrative proxy for various farming practices.

## 3 RESULTS

### 3.1 Is predation on *D. gallinae* by naturally occurring mites possible?

**FIGURE 2:**
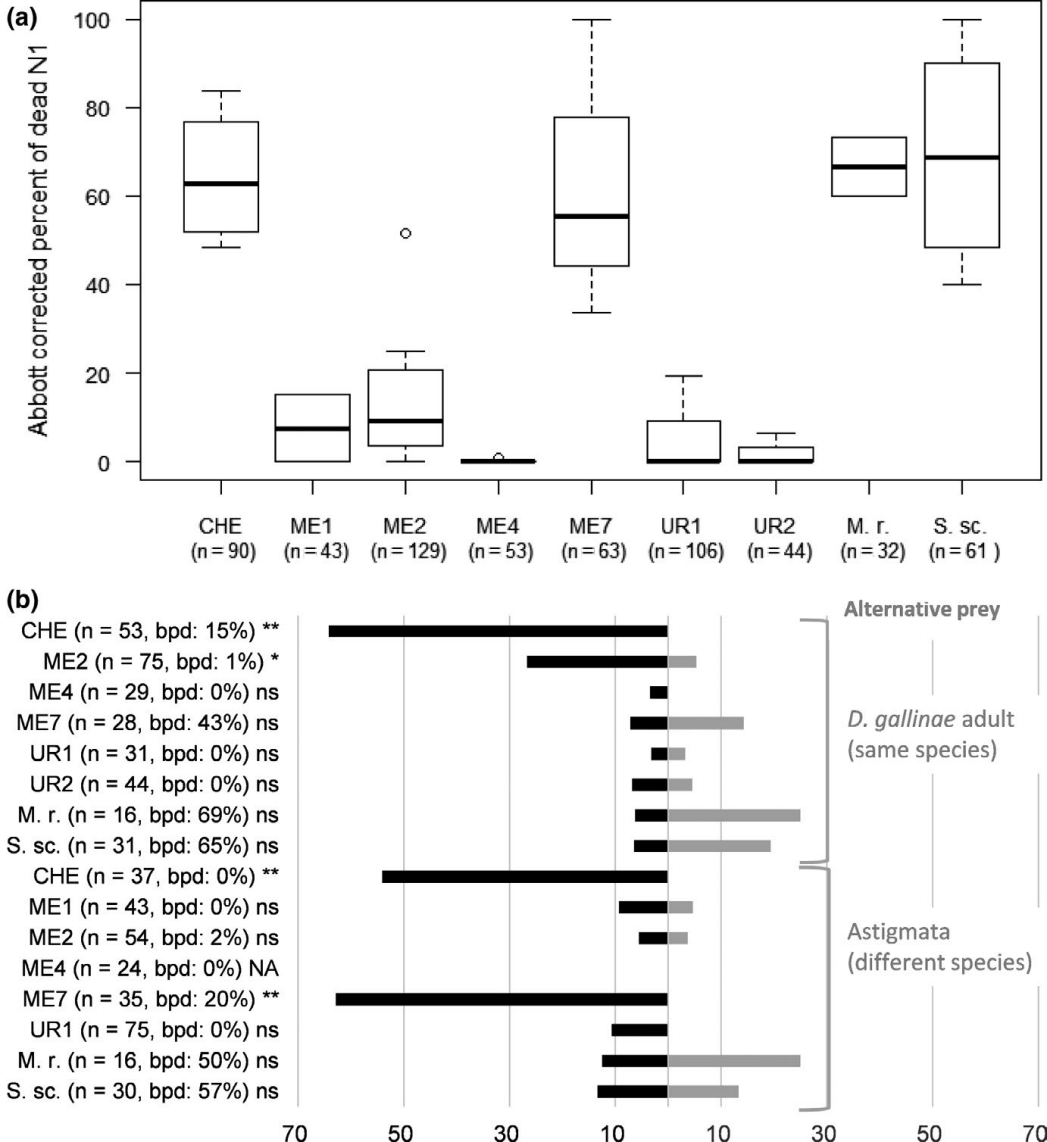
In vitro predation bioassays. (a) Boxplot of Abbott‐corrected percentage of mortality of *Dermanyssus gallinae* protonymphs according to predator. (b) Distribution of choices according to predator and alternative prey (uncorrected % of all records). Here, only wells with a single prey dead are figured (either *D. gallinae* protonymphs or alternative prey). Morphospecies ID as in Table 1; S. sc., *Stratiolaelaps* *scimitus* and M. r., *Macrocheles robustulus* (control generalist predators); *n*, number of predatory mites under test; black horizontal bars, % of wells with *D. gallinae* protonymph killed; grey horizontal bars, % of wells with alternative preys killed; bpd (= both preys dead), percentage of wells where both prey were dead; signs following the parentheses, significance according to binomial exact test on choices: ns, not significant, *p < .05, **p < .01, ***p < .001

The in vitro test system was considered correct since we recorded high frequencies of predation events on all mite preys (>80% wells with predation) and no significant preference according to the stage or the taxon with the control generalist predators (*M. robustulus* and *S. scimitus*; Figure 2).

*Cheyletus* spp. (CHE) and *M. muscaedomesticae* (ME7) had high predation frequencies on *D. gallinae* protonymphs (close to generalist controls; Figure 2a)*. Androlaelaps casalis* (ME2) and Digamasellidae spp. (ME1) had overall low predation frequency. Uropodina (UR1 and UR2) and *Proctolaelaps parascolyti* (ME4) preyed only anecdotally on any mite or were not reported to do so*. Androlaelaps casalis* (ME2) exhibited high variation in predation rate, with one record above 50% of predation in spite of a median below 10% (Figure 2a). Overall, our experimental tests revealed that the following three taxa are capable of substantial predation on *D. gallinae* in the absence of any physical barrier: *Cheyletus* spp. (CHE), *M. muscaedomesticae* (ME7) and *A. casalis* (ME2). They will be referred to as “pest predators” in the remaining text.

*Androlaelaps casalis* favored protonymphs (Figure 2b). *Cheyletus* spp. (CHE) and *M. muscaedomesticae* (ME7) both preferred significantly *D. gallinae* (any stage) versus Astigmata (Figure 2b). Predation on *D. gallinae* protonymphs by *A. casalis* was reduced in the presence of Astigmatid mites (5.6%) compared to adult *D. gallinae* (26.7%).

### 3.2 Can predation take place in real life?

Assessment of the quality of air DNA analyses is provided in Appendix S2. Metagenomic data had satisfactory sensitivity and specificity: First, the selected DNA fragment allowed to distinguish all of the morphospecies except two (same sequence for the morphospecies ME1 and ME4); second, we recorded OTUs of arthropods previously observed by the naked eye in the high stratum only (e.g., spiders, moths, staphylinid beetles); lastly, the number of sequences of *D. gallinae* in the air were well correlated with the number of individuals counted in manure (Spearman's *ρ* = 0.56). This makes us confident that the two indicators (airborne DNA and mite counts from manure) of the focus prey are good proxy to estimate the variations of pest populations in henhouses, a critical point when working on the abundance of *D. gallinae* in henhouses (see Mul et al., 2017, 2015).

The relative abundances of *D. gallinae* assessed through airborne DNA were positively correlated with the relative abundances of the three predator taxa identified as predators during experimental tests (*Cheyletus* spp.,* ρ *= 0.31, *Macrocheles* spp.,* ρ *= 0.31, *A. casalis, ρ *= 0.41). None of the other potential predators (other mites, spiders, Pseudoscorpionida, bugs) correlated with *D. gallinae* in airborne DNA. However, we also found significant positive correlation between *D. gallinae* and Astigmatid mites (*ρ *= 0.62, *p* = .0001). Astigmatid mites are strictly detritivorous and incapable of predation. Looking at the air and manure data, the three pest predators were very common locally (manure sample) and almost ubiquitous at the barn level (Table 2).

**TABLE 2:** Percentage of detected occurrence of the three predators of *Dermanyssus gallinae* from manure and air data

	Per sample (manure data; *n* = 315)	Per henhouse × campaign (manure data; *n* = 63)	Per henhouse × campaign (manure and air data; *n* = 68)
*Androlaelaps casalis* (ME2)	41.90%	76.19%	86.76%
*Macrocheles* spp. (ME7)	53.97%	88.89%	85.29%
*Cheyletus* spp. (CHE)	40.63%	68.25%	95.59%

### 3.3 Does this predation differ from one practice to another?

**FIGURE 3:**
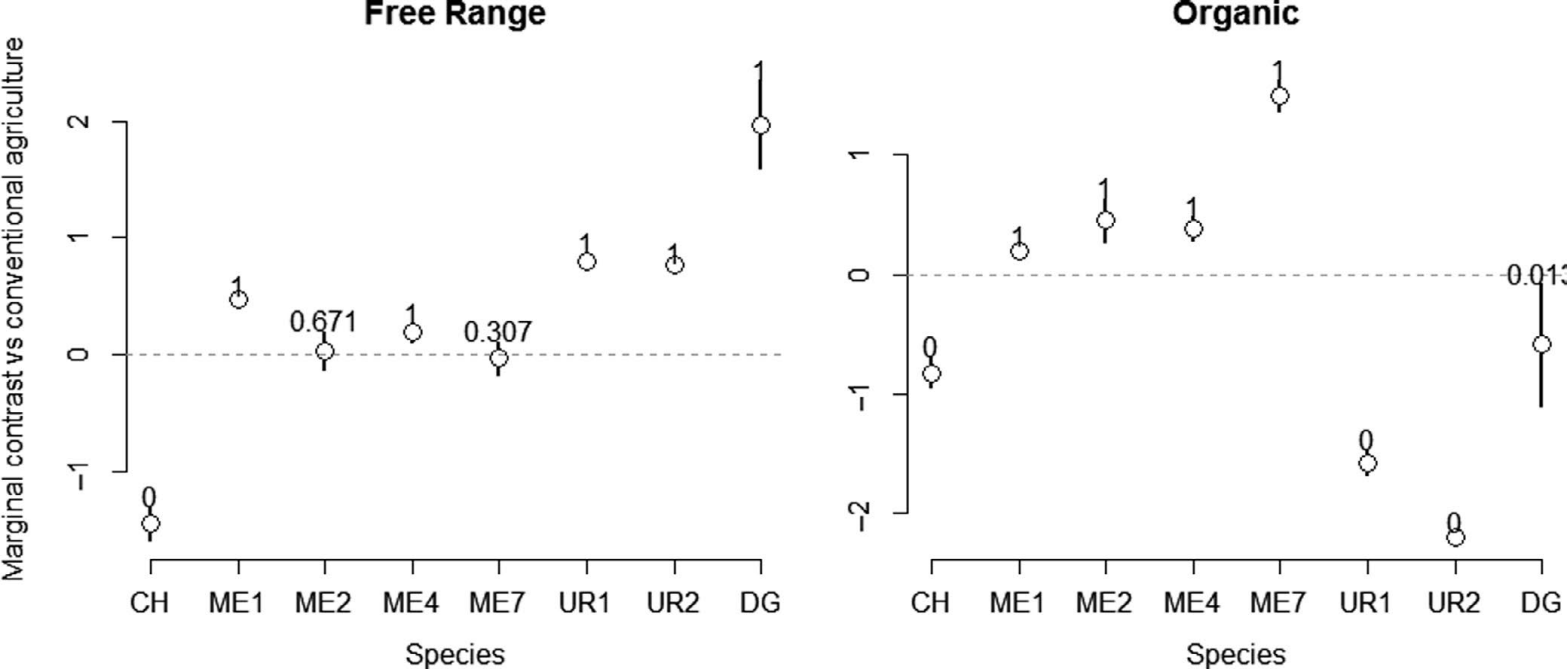
Posterior densities of the morphospecies abundance (log) contrast between types of farm management with the conventional type as a reference

The fit of our hierarchical model was adequate, as assessed by a Bayesian posterior predictive check (Appendix S3). As expected from the exploration of count distributions, these checks did not reveal any major impact of count overdispersion. Farm type had a strong effect on mite counts in manure, but with considerable variation in sign and magnitude. Most morphospecies were more numerous in organic and to a lesser extent in free‐range than in conventional farms, with the exception of *Cheyletus* spp. (CHE), Uropodina (UR1 and UR2) and *D. gallinae* (Figure 3). Counts of *Cheyletus* spp. (CHE) and of the Uropodine morphospecies (UR1 and UR2) were higher in conventional farms than in organic farms. Despite high variation, counts of *D. gallinae* (DG) in manure samples were higher in free‐range and lower in organic farms than in conventional farms (Figure 3).

**FIGURE 4:**
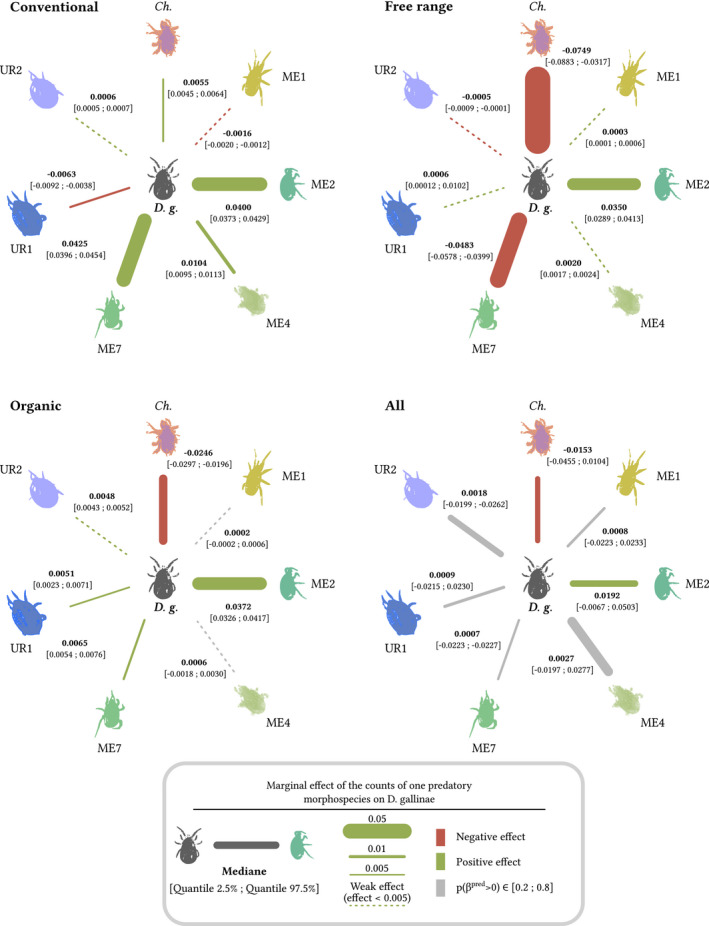
Prey–predator covariations as inferred from the Bayesian hierarchical model

Conforming to our experimental conclusions, our model revealed covariations between *D. gallinae* and the three mites identified as predators in in vitro predation tests, but not with any other morphospecies. Covariations were constantly positive between *D. gallinae* and *A. casalis,* but alternated between positive and negative for the two other predators (Figure 4). Irrespective of farm type, counts of *D. gallinae* were positively correlated with counts of *A. casalis* (ME2) (*p*(*β*_ME2_> 0) = 0.914) and negatively correlated with counts of *Cheyletus* sp. (CHE) (*p*(*β*_CHE_> 0) = 0.145) (Figure 4, “all types”). Within each of the three farm types, counts of *D. gallinae* were positively correlated with counts of *A. casalis* (ME2) (*p*(*β*_ME2_> 0) = 1), but their correlation with counts of *Cheyletus* spp. (CHE) and of *Macrocheles* spp. (ME7) depended on farm type (*p*(*β*_CHE_> 0) = 1 in conventional farms, and *p*(*β*_CHE_> 0) = 0 elsewhere; *p*(*β*_ME7_> 0) = 0 in free‐range farms, *p*(*β*_ME7_> 0) = 1 elsewhere). Weak or null effects were associated with all other combinations of type of farm management and morphospecies.

The other covariates also had a strong effect (Appendix S3): Flock age was associated with higher counts of all morphospecies but ME2 and ME4 (*p*(*β*_flock_ > 0) = 0 for ME4, 0.48 for ME2 and 1 for all others). The gamasine mesostigmatic mites (ME1, ME2, ME4, ME7) were less numerous in the Ain than in the Rhône (*p*(*β*_Ain_ > 0) = 0), while *Cheyletus* spp. and uropodine mites exhibited the inverse pattern (*p*(*β*_Ain_ > 0) = 1). Counts peaked in spring and summer, followed with a decrease in autumn in *P. parascolyti* (ME4), *Macrocheles* spp. (ME7), Uropodina (UR2) and *D. gallinae* (Appendix S3). *Cheyletus* spp. (CHE) exhibited an inverse dynamic, with a peak in autumn, followed by a sharp decline in winter‐spring, then a slight increase in summer. Seasonal variations were lower in ME1, ME2 and UR1. All *p*(*β*_Season_ > 0) were either <0.01 or >0.99 (Appendix S3).

## 4 DISCUSSION

We provided unique empirical evidence for trophic interactions in a mite assemblage compatible with a local regulating effect on pest populations within livestock farms. The combination of in vitro predation tests and correlative analyses supported an assemblage‐level effect of predation on the targeted hen parasite, *D. gallinae,* by three mite predators. Farming practices did not, however, seem to affect consistently this pattern. Even if our results do not demonstrate the existence of a biological control service in livestock farms, they should call attention toward the possible emergence of ignored spontaneous biological control services in commercial livestock farms.

In our study system, the pest control service results from predation among organisms that are difficult to study because of their tiny size and their association with vertebrates. Studying a predation process in this context was challenging because no in situ observations or experiments can be done in the same way as for phytophagous insects: as a hematophagous species, *D. gallinae* is too fragile and too mobile to be subjected to controlled designs in the field. Predator exclusion is also impracticable because predators and pests are similar in size. Furthermore, signs of damage induced by the pest are undetectable since no visible skin lesions are induced by *D. gallinae* on hens. In the present study, we bypassed these difficulties by combining correlative approaches and an in vitro experiment, complemented by inventories based on different techniques. Even if our study did not produce clear‐cut answers to our initial questions due to the impossibility of any direct observation of the process of interest, we have truly explored prey–predator interactions in the poultry mite world for the first time.

### 4.1 Possible explanations for the over‐representation of positive prey–predator covariations

Two groups of explanations can be considered, one linked to the dynamics and demography of the species under consideration, the other to a methodological bias. First, the predation observed in vitro did not translate in the field through simple negative prey–predator regulating dynamics. Instead, most correlations between the prey and its predators were positive, implying either that the prey population is limited more by the resource (bottom‐up) than by predation (top‐down), or that predation has a positive influence on the prey population. The limitation of the prey population by the resource largely dominates the limitation by predation at different trophic levels in some aquatic systems (e.g., Osenberg & Mittelbach, 1996). If the same is true for our system, positive correlations between predator and prey are therefore compatible with the actual occurrence of predation under the nonlimiting resource conditions that prevail in farms. However, this imbalance between bottom‐up and top‐down limitation is not so obvious in terrestrial systems (Krebs, Boonstra, Boutin, & Sinclair, 2001). The predation of *Cheyletus* spp. by *A. casalis* observed in the laboratory (Zriki et al., 2020) may induce a cascade effect in henhouses, which could result in a positive effect of *A. casalis* on *D. gallinae* (Abrams, 1992). Furthermore, the antibodies that are produced by hens in response to native *D. gallinae* antigens significantly reduce the mite population growth (Bartley et al., 2017). Thus, *D. gallinae* likely has a direct negative effect on its own growth rate per individual, and predation at a key point in this dynamics could increase equilibrium pest density (Abrams, 1992) by inhibiting antibody production.

Positive prey–predator covariations can alternatively be explained by the omnipresence of predators in our sample. The expected negative covariation between a regulating predator and its prey should result from a limitation in the temporal dynamic of the prey by the predation process (Osenberg & Mittelbach, 1996). In our correlative field study, we measure prey–predator covariations on the basis of local prey and predator populations sampled in space and time, but without time series (different sampling points in each campaign). Therefore, the expected negative covariation between effective predator and prey can only be measured if both predator‐exposed and unexposed populations of *D. gallinae* are represented in the dataset. The frequency of predators in field samples determines the possibility of detecting a negative effect of the predation process: consistent with Burtt, Chow, and Babbitt (1991), strictly positive prey–predator covariations are expected in datasets where the predator is omnipresent (as is the case here for *A. casalis*). Opposite to our results, but consistent with this, a significant negative covariation was reported between *Androlaelaps casalis* and *Dermanyssus gallinae* in starling nestboxes (Lesna et al., 2009). Since *A. casalis* is an active hunter, moving individually for several meters in search of prey (Barker, 1968), the fragmented environment provided by nestbox‐based colonies may impair its dispersal. As a result, the layout of bird colonies in nesting boxes may trigger heterogeneity in predation pressure on *D. gallinae*, while the layout of poultry houses allows for homogeneous predation pressure. This contrast explains well why we found an opposite correlation between the same two species in our more connected study system. Hence, the positive prey–predator covariations that we observed do not infirm the possibility of a regulating service in henhouses, although our data did not allow to demonstrate its existence or absence. A similar conclusion was drawn from spider‐insect positive covariations (Cotes et al. 2018) and exclusion experiments showed that ignored biological control is exerted by naturally occurring enemies in crops (e.g., Dainese, Schneider, Krauss, & Steffan‐Dewenter, 2017; Librán‐Embid et al., 2017; Rusch, Bommarco, Jonsson, Smith, & Ekbom, 2013). The impact of predation may alternatively emerge as a result of assemblages dynamics, although the ubiquity of predators impairs its species‐level identification. Since exclusion of mites in poultry houses is not feasible, an efficient, yet costly improvement would aim to compare the evolution of *D. gallinae* populations in the presence or absence of predators in mite‐proof isolators equipped to house hens for several generations of mites.

### 4.2 No consistent signal about the positive effect of organic practices on the predation process

Contrary to our predictions, organic practices did not seem to foster mite predation on *D. gallinae* despite the absence of synthetic insecticide and the observed higher mite diversity. The effects of farm type on pest–predator interactions were unclear overall, although substantial negative covariations were limited to organic, as expected, and free‐range farms. These obscure patterns are consistent with previous correlative studies at similar scales on crop production systems (e.g., Bengtsson et al., 2005; Paoletti et al., 1995; Winqvist et al., 2011). The idiosyncratic directions of prey–predator covariations could be a consequence of food web complexity and species’ independent variations (Karp et al., 2018; Librán‐Embid et al., 2017; Paine, Tegner, & Johnson, 1998). The rare cases of negative covariation could result from a less homogeneous distribution of the predators concerned, which would simply make it possible to detect differences in the demographic development of *D. gallinae* between points with a predator and points without any predator. More specifically the organic farms may contain points with and without *Cheyletus* spp. with effectively dampened *D. gallinae* dynamics in the second group. This is consistent with the lower number of *Cheyletus* spp. counted in these farms compared to other farm types. Free‐range farms may contain more points where *Macrocheles* spp. is absent than other farm types, but this species did not seem to be less abundant in free‐range than in conventional farms. The negative covariation that we observed in free‐range farms between *D. gallinae* and *Macrocheles* spp. could also result from *M. muscaedomesticae* feeding disproportionately more on *D. gallinae* when its preferred preys, nematodes, are less abundant (prey switching, Murdoch, 1969). Synthetic deworming treatments are almost exclusively and systematically applied in free‐range farms and the only deworming molecule applied in the present free‐range farms is harmless to arthropods (benzimidazoles; Lumaret & Errouissi, 2002). Prey switching from nematodes to mites may thus be an unintended beneficial consequence of a farming practice (deworming in free‐range) which indirectly contributes to regulate *D. gallinae*. Similarly, if the methodological bias related to the ubiquity of predators is not the reason for the lack of detected effects, a disruption of the natural pest control service due to the use of insecticides (Mohammed et al., 2017; Tscharntke et al., 2016) in conventional farms could partly explain the lack of negative prey–predator covariation. Anti‐fly neurotoxic insecticides are used in conventional farms and neurotoxic insecticides may reduce the ability of arachnid predators to catch preys (Řezáč, Řezáčová, & Heneberg, 2019). Neurotoxic anti‐fly insecticides are, however, also used in free‐range farms. In conclusion, the farming practices tested here do not have a strong enough effect to unambiguously affect prey–predator interactions at the poultry house level, or interactions with various factors not studied explain the contradictions found.

## 5 CONCLUSION

Our study provided evidence for predation by mites on a major parasite in poultry farms, but this process did not unambiguously translate into an assemblage‐level signal, which prevents us from inferring a major pest regulation service. This conclusion comes with caution however, since multiple predation‐compatible processes may explain many of the covariations that we observed at the assemblage‐scale. In order to clearly detect the footprint of a service in our system where predators are pervasive, we must go through more experimental approaches that would bypass limitations of correlative studies. Terrestrial mesocosms such as those of soil arthropods (e.g., Cortet et al., 2006; D'Annibale et al., 2015) would be good tools to assess the suppressive effect of different predators on *D. gallinae*. On the whole, a good knowledge of the functional ecology of the assemblages surrounding the farming system is crucial to stack promising and productive ecosystem services into an operational, profitable and sustainable whole (“ecostacking” sensu Hokkanen, 2017). Our results demonstrate the necessity of advancing the understanding of trophic interactions among small organisms to achieve coherent ecological intensification of agriculture and reduce massive manure‐driven environmental pollution.

## CONFLICT OF INTEREST

The authors declare that there is no conflict of interest.

## AUTHOR CONTRIBUTION

**Lise Roy:** Conceptualization (lead); Data curation (equal); Formal analysis (equal); Funding acquisition (lead); Methodology (equal); Project administration (equal); Supervision (lead); Visualization (equal); Writing‐original draft (lead); Writing‐review & editing (equal). **Adrien Taudière:** Data curation (equal); Formal analysis (equal); Methodology (equal); Software (equal); Validation (equal); Visualization (equal); Writing‐review & editing (equal). **Julien Papaix:** Formal analysis (equal); Validation (equal); Writing‐review & editing (equal). **Rumsais Blatrix:** Formal analysis (equal); Investigation (equal); Writing‐review & editing (equal). **Geoffrey Chiron:** Conceptualization (equal); Funding acquisition (lead); Project administration (equal); Writing‐review & editing (equal). **Ghais Zriki:** Investigation (equal); Writing‐review & editing (equal). **Olivier Bonato:** Conceptualization (equal); Writing‐review & editing (equal). **Jean‐Yves Barnagaud:** Formal analysis (equal); Investigation (equal); Software (equal); Validation (equal); Writing‐original draft (lead); Writing‐review & editing (equal).

## Supporting information

Appendix S1

Appendix S2

Appendix S3

## Data Availability

Data on airborne DNA sequences (ILLUMINA) have been deposited on Dryad (https://doi.org/10.5061/dryad.nzs7h44pf). This dataset is mentioned in Sections 2.1.3 and 2.3 of the Materials and Methods section and has been used in the Results section to answer the question “Can predation occur in real life?”. Mite morphospecies count data from manure per sample with farm, management type, sampling season, and flock age information have been deposited on Dryad (doi: https://doi.org/10.5061/dryad.ghx3ffbkz). This dataset is mentioned in Sections 2.1.3, 2.3 and 2.4 of the Materials and Methods section and was used in the Results section to answer the question “Does this predation differ from one practice to another?” In order to refine the taxonomic assignment of air DNA data, this dataset has also been cross‐referenced with the previous dataset and with Sanger sequencing data obtained on the same mites in the framework (see end of Section 2.3).
