# Supplementary material for: Evaluating the link between predation and pest control services in the mite world
Source: Ecol Evol. 2020 Aug 15;10(18):9968–80. doi: 10.1002/ece3.6655 (PMC7520221; doi:10.1002/ece3.6655)
Supplement: Supplementary file 1 — Appendix S1 [file ECE3-10-9968-s001.pdf]

Supplementary material S1. Information of henhouses and farming practices.

Henhouse ID	Type of farm management	Region	Open-air access available	Hen density at the start of flock (hens /m^2)	Regular deworming treatment (every 5-6 weeks)	Recorded deworming active substances	Recorded treatments during empty period				Recorded treatment during flock (sampling campaigns 1+2+3)							
							pyrethroid (approved)	disinfectant	flora	quicklime	pyrethroid (not approved)	organophosphate (approved)	cyromazin	grease in PRM hiding places	soap or soapy water	disinfectant	wasp (against flies)	Predatory mite against PRM
F1	organic	Ain	yes	5,1	yes	plant extracts …		x						x	x			
F10	free-range	Ain	yes	9,0	yes	flubendazole		x				x						
F11	organic	Drôme (exten	yes	6,0	no	plant extracts …		x							x	x		
F12	free-range	Ain	yes	7,5	no	-		x				x						
F13	conventional	Ain	no	8,4	no	-	x	x						x				
F14a	conventional	Ain	no	8,9	no	-	x	x	x		?	?						
F14b	conventional	Ain	no	8,6	no	-	x	x	x		?	?		x				
F15	free-range	Ain	yes	7,5	no	-	x				?							
F16	organic	Drôme (exten	yes	6,0	yes	plant extracts …		x		x					x			
F2	conventional	Drôme (exten	no	7,3	yes	flubendazole	x	x	x							x		
F3a	free range	Drôme (exten	yes	9,7	yes	flubendazole						x	x					
F3b	free range	Drôme (exten	yes	9,7	yes	flubendazole						x	x					
F4	free range	Drôme (exten	yes	8,9	yes	flubendazole		x						x			x	
F5a	organic	Drôme (exten	yes	5,6	yes	plant extracts …		?	x	x				x			x	
F5b	organic	Drôme (exten	yes	6,5	yes	plant extracts …		?	x	x				x			x	
F6a	conventional	Ain	no	9,0	no	-	x	x						x				
F6b	conventional	Ain	no	9,2	no	-	x	x						x				
F7	free range	Drôme (exten	yes	9,3	no	-		x										
F8	organic	Drôme (exten	yes	4,5	yes	plant extracts …		x	x									
F9	free range	Ain	yes	10,3	yes	flubendazole	x	x						x				x

"Ain" is a French department. The “Drôme (extend.)” area is located mainly in the Drôme department, with some extension in adjacent departments (one farm in the Gard, one in the Vaucluse)
