# Supplementary material for: Evaluating the link between predation and pest control services in the mite world
Source: Ecol Evol. 2020 Aug 15;10(18):9968–80. doi: 10.1002/ece3.6655 (PMC7520221; doi:10.1002/ece3.6655)
Supplement: Supplementary file 2 — Appendix S2 [file ECE3-10-9968-s002.pdf]

Supplementary material S2.

Information on mite communities from airborne DNA

Bioinformatics pipeline

See page 3.

Sensitivity and specificity

To assess our Illumina sequencing data, we checked the sensitivity of the method for mite inventories by (1) checking whether the DNA sequences obtained from a subsample of manure-dwelling mites recorded during the study were present in the Illumina dataset, (2) estimating the discriminating power of the 18S fragment using the present pipeline by checking whether the assignment of the sequences to taxa corresponding to the different mosphospecies was correct, (3) measuring correlation between air Illumina and manure visual records per henhouse. We also estimated the sensitivity of the detection of the different morphospecies in airborne DNA by (4) comparing molecular and visual occurrences in the different farms. Here are the obtained results:

(1) For all mite morphospecies, including the seven predatory morphospecies and D. gallinae under test here, plus ME5 (excluded from the present tests as unfrequent) and Astigmatid mites (typically detritivorous and abundant mites), the corresponding Sanger-sequenced DNA fragments from Roy et al. (2017) were effectively present within the Illumina dataset.

(2) The bioinformatics pipeline resulted in their assignment to distinct OTUs with consistent taxonomic assignment, except for ME1 and ME4. The discriminating power of the selected DNA fragment was satisfying as it allowed to distinguish all of the morphospecies except two (same sequences for ME1 and ME4). The latter two were both shown not to directly interact with *D. gallinae* (null effect size and absence of *in-vitro* predation), so this lack of specificity is considered devoid of any consequence here.

(3) This was supported by the significant positive Spearmann rho values obtained with molecular and visual counts of five mite morphospecies (*Cheyletus sp.,* ME2, ME7, UR1, *D. gallinae*) and several other arthropod species (below table). The absence of any

significant correlation with a few morphospecies may be explained by either too much rare occurrence and preferred habitat different from poultry manure (ME5, Oribatida) or discrepancies in terms of sequencing efficiency (UR1, UR2).

Table. Comparison between molecular and visual counts (from manure samples) of mite morphospecies. The first four columns depict the number of samples where each morphospecies was found (i) both molecularly and visually, (ii) neither molecularly nor visually, (iii) only molecularly and (iv) only visually. The *rho* column indicates the strength of the correlation of molecular abundance and visual abundance using the non-parametric Spearman rank test. P, associated p-value from the Spearman rank test.

	**Double presence**	**Double absence**	**Molecular detection (air) only**	**Visual detection (manure) only**	***rho***	**p**
Astigmata	67	0	0	1	0.329	0.00634
Cheyletus spp.	39	3	19	7	0.317	0.00847
D. gallinae (PRM)	67	0	1	0	0.575	0.00001
ME1 + ME4	66	0	0	2	-0.034	0.78048
ME2	30	9	9	20	0.384	0.00123
ME5	4	45	13	6	0.158	0.19714
ME7	18	10	1	39	0.291	0.01611
Oribatida	1	48	18	1	0.129	0.29433
UR1	16	10	1	41	0.495	0.00002
UR2	2	2	0	64	0.232	0.05735

(4) In terms of sensitivity of air DNA analyses, a satisfying detection power for *Cheyletus,* ME1+ME4, ME5, as well as most of other arthropod morphospecies was recorded, with the majority of visual and molecular records concordant in terms of presence/absence assessment. Some failures to molecularly detect ME2, ME7 and UR1 from airborne particles were recorded (20/59, 39/58 and 41/58 positive samples, resp.).

An overall satisfying sensitivity and specificity of the 18S sequencing from airborne DNA as well as a good concordance between the different record means (mite individuals from manure and DNA sequences from airborne particles) were observed.

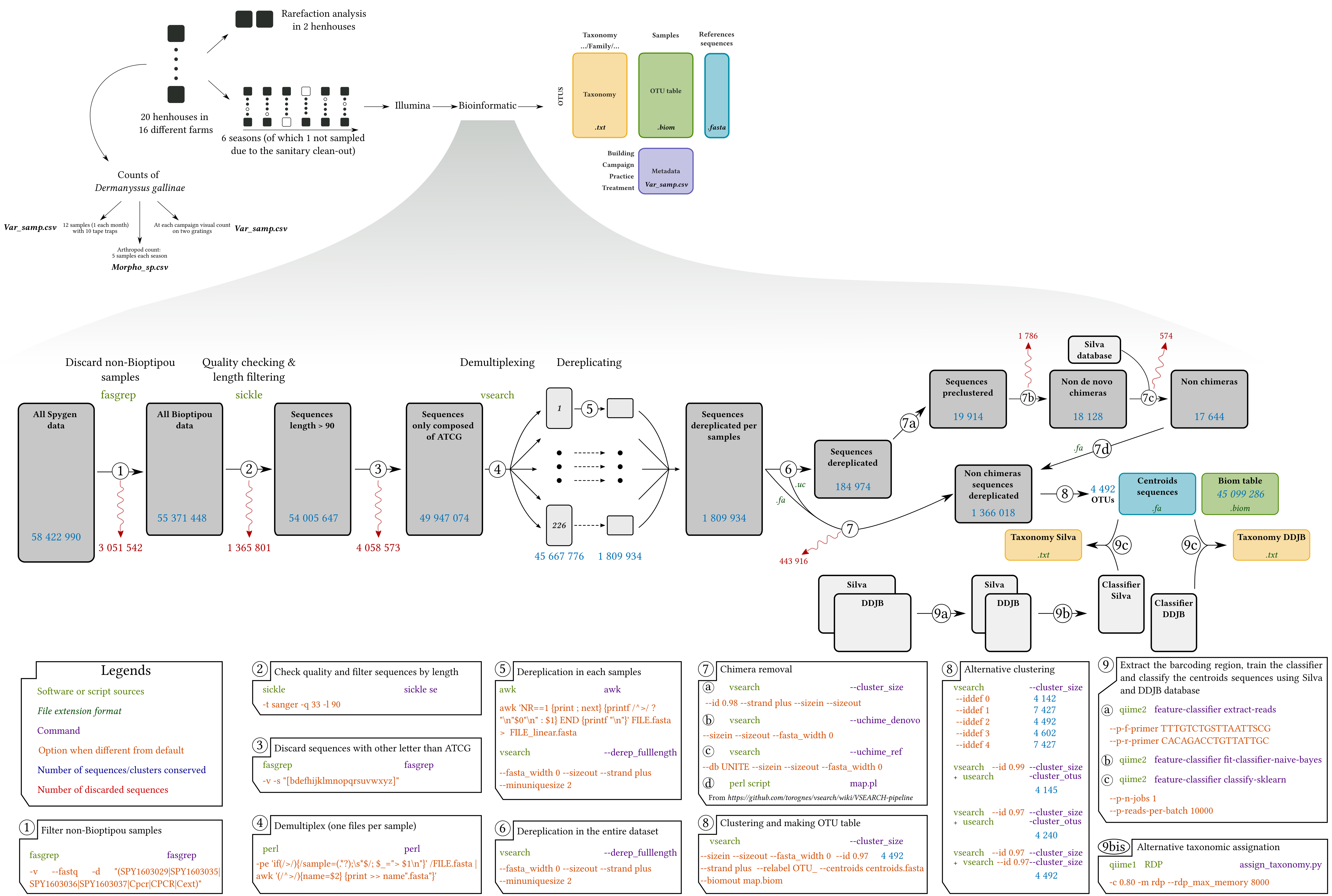

Rarefaction analysis in 2 henhouses
20 henhouses in 16 different farms
6 seasons (of which 1 not sampled due to the sanitary clean-out)
Illumina
Bioinformatic
Counts of Dermanyssus gallinae
Var_samp.csv
12 samples (1 each month) with 10 tape traps
At each campaign visual count on two gratings
Var_samp.csv
Arthropod count: 5 samples each season
Morpho_sp.csv
Taxonomy .../Family/...
Samples
References sequences
OTUS
Taxonomy .txt
OTU table .biom
.fasta
Building
Campaign
Practice
Treatment
Metadata Var_samp.csv
Discard non-Bioptipou samples
fasgrep
Quality checking & length filtering
sickle
Demultiplexing
vsearch
Dereplicating
All Spygen data 58 422 990
3 051 542
All Bioptipou data 55 371 448
1 365 801
Sequences length > 90 54 005 647
4 058 573
Sequences only composed of ATCG 49 947 074
1
226
45 667 776
1 809 934
Sequences dereplicated per samples 1 809 934
.uc
.fa
Sequences dereplicated 184 974
443 916
Sequences preclustered 19 914
1 786
Silva database
Non de novo chimeras 18 128
574
Non chimeras 17 644
.fa
Non chimeras sequences dereplicated 1 366 018
4 492 OTUs
Centroids sequences .fa
Biom table 45 099 286 .biom
Taxonomy Silva .txt
Taxonomy DDJB .txt
Silva
DDJB
Classifier Silva
Classifier DDJB
Legends
Software or script sources
File extension format
Command
Option when different from default
Number of sequences/clusters conserved
Number of discarded sequences
1 Filter non-Bioptipou samples
fasgrep fasgrep
-v --fastq -d "(SPY1603029|SPY1603035|SPY1603036|SPY1603037|Cpcr|CPCR|Cext)"
2 Check quality and filter sequences by length
sickle sickle se
-t sanger -q 33 -l 90
3 Discard sequences with other letter than ATCG
fasgrep fasgrep
-v -s "[bdefhijklmnopqrsuvwxyz]"
4 Demultiplex (one files per sample)
perl perl
-pe 'if(/>/){/sample=(.*?);\s*$/; $_="> $1\n"}' /FILE.fasta | awk '(/^>/){name=$2} {print >> name".fasta"}'
5 Dereplication in each samples
awk awk
awk 'NR==1 {print ; next} {printf /^>/ ? "\n"$0"\n" : $1} END {printf "\n"}' FILE.fasta > FILE_linear.fasta
vsearch --derep_fulllength
--fasta_width 0 --sizeout --strand plus --minuniquesize 2
6 Dereplication in the entire dataset
vsearch --derep_fulllength
--fasta_width 0 --sizeout --strand plus --minuniquesize 2
7 Chimera removal
a vsearch --cluster_size
--id 0.98 --strand plus --sizein --sizeout
b vsearch --uchime_denovo
--sizein --sizeout --fasta_width 0
c vsearch --uchime_ref
--db UNITE --sizein --sizeout --fasta_width 0
d perl script map.pl
From https://github.com/torognes/vsearch/wiki/VSEARCH-pipeline
8 Clustering and making OTU table
vsearch --cluster_size
--sizein --sizeout --fasta_width 0 --id 0.97 4 492
--strand plus --relabel OTU_ --centroids centroids.fasta
--biomout map.biom
8 Alternative clustering
vsearch --cluster_size
--iddef 0 4 142
--iddef 1 7 427
--iddef 2 4 492
--iddef 3 4 602
--iddef 4 7 427
vsearch --id 0.99 --cluster_size + usearch -cluster_otus 4 145
vsearch --id 0.97 --cluster_size + usearch -cluster_otus 4 240
vsearch --id 0.97 --cluster_size + vsearch --id 0.97 --cluster_size 4 492
9 Extract the barcoding region, train the classifier and classify the centroids sequences using Silva and DDJB database
a qiime2 feature-classifier extract-reads
--p-f-primer TTTGTCTGSTTAATTSCG
--p-r-primer CACAGACCTGTTATTGC
b qiime2 feature-classifier fit-classifier-naive-bayes
c qiime2 feature-classifier classify-sklearn
--p-n-jobs 1
--p-reads-per-batch 10000
9bis Alternative taxonomic assignation
qiime1 RDP assign_taxonomy.py
-c 0.80 -m rdp --rdp_max_memory 8000
