# Supplementary material for: Evaluating the link between predation and pest control services in the mite world
Source: Ecol Evol. 2020 Aug 15;10(18):9968–80. doi: 10.1002/ece3.6655 (PMC7520221; doi:10.1002/ece3.6655)
Supplement: Supplementary file 3 — Appendix S3 [file ECE3-10-9968-s003.pdf]

Supplementary material S3. Detailed description of our Bayesian model, fit assessment and effect of remaining covariates from its outputs (manure data).

Model description

We developed a hierarchical Bayesian model to estimate covariations between the abundance of seven putative predators and the abundance of *D. gallinae* from manure records.

We first summed mite counts per morphospecies, henhouse (5 samples of 250 mL manure per henhouse), and sampling campaign (4), and considered it as a proxy of predator abundance Npred per sample i, predator morphospecies s and season k. We further assumed that Npred was an exact count since variations in detectability are not adequately estimated with overly dispersed Poisson data (Knape et al., 2018). Due to the low probability of detecting mites present in small numbers in coarse substrates, we used a method of extracting mites designed to optimize mite recovery from poultry manure (Roy et al., 2017) and considered that the proportions of mites correctly represented community composition. Hence, $Npred_{i,s,k} \sim P\left(\lambda_{i,s,k}^{\text{pred}}\right)$ where the intensity $\lambda_{i,s,k}^{\text{pred}}$ was a log-linear function of species-specific effects of farm type (conventional, free-range or organic), flock age, season and region, with a species-specific intercept α and linear coefficients β:

$$log\left(\lambda_{i,s,k}^{\text{pred}}\right) = \alpha_s^{\text{pred}} + \beta_{s,\text{farmtype}(i)}^{(1)} + \beta_s^{(2)} * flockage_{i,k} + \beta_{s,k}^{(3)} + \beta_{s,\text{region}(i)}^{(4)}$$

We then modelled *D. gallinae* abundance, Nprey, in a sample i and a season k as a Poisson distribution with intensity $\lambda_{i,k}^{\text{prey}}$, a log-linear function of predator abundances, farm type, flock age and region:

$$Nprey_{i,k} \sim P\left(\lambda_{i,k}^{\text{prey}}\right)$$

$$log\left(\lambda_{i,k}^{\text{prey}}\right) = \alpha_{\text{farmtype}(i)}^{\text{prey}} + \sum_{s=1}^{S}\left(\gamma_{s,\text{farmtype}(i)}^{(1)} \times \lambda_{i,s,k}^{\text{pred}}\right) + \gamma^{(2)} \times flockage_{i,k} + \gamma_k^{(3)} + \gamma_{\text{region}(i)}^{(4)}$$

We assigned hierarchical hyperpriors to $\gamma^{(1)}$ with common means μ_s per predator species and a common variance σ, in order to estimate predator-specific effects on *D. gallinae* abundance irrespective of farm type:

$$\gamma_{s,\text{farmtype}(i)}^{(1)} \sim N(\mu_s, \sigma)$$

We then assigned a hierarchical hyperprior to μ_s with common mean $\bar{\mu}$ across all species and common variance σ', in order to estimate the overall (mean) effect of predators on the prey:

$$\mu_s \sim N(\bar{\mu}, \sigma')$$

We fixed $\alpha^{\text{pred}}_{\text{conventional farms}}$, $\alpha^{\text{prey}}_{\text{conventional farms}}$, $\beta^{(3)}_{\text{autumn}}$, $\gamma^{(3)}_{\text{autumn}}$, $\beta^{(4)}_{\text{Ain}}$, $\gamma^{(4)}_{\text{Ain}}$ to 0 to ensure model identifiability. We specified non-informative normal priors to mean parameters (N(0,1000)) and uniform priors to the inverse of variance parameters (U(0,100)).

We estimated the model parameters with Monte-Carlo-Markov-Chain algorithms under Jags 3.1.0 (Plummer, 2003).

Assessment of the fit

We ran 3 chains of 40 000 iterations, discarding the 20 000 first as a burn-in and thinning by 20. Gelman's Rhat (Gelman, 2014) and visual chain exploration revealed adequate parameter convergence (Rhat < 1.1 for all parameters; see table S3-1). We evaluated fit by comparing observed Npred or Nprey to values replicated by the model through a Bayesian posterior predictive check (Fig. S3-1, S3-2).

In a Bayesian framework, parameters uncertainty is assessed with empirical credible intervals corresponding to empirical 2.5% and 97.5% quantiles computed on the MCMC samples. We used Bayesian p-values to assess the extent to which each parameter departed from 0 ; these p-values are continuous and computed as frequencies (p = Number of iterations > 0 / MCMC chain length, hence p = 0.5 implies that the parameter is centered on 0).

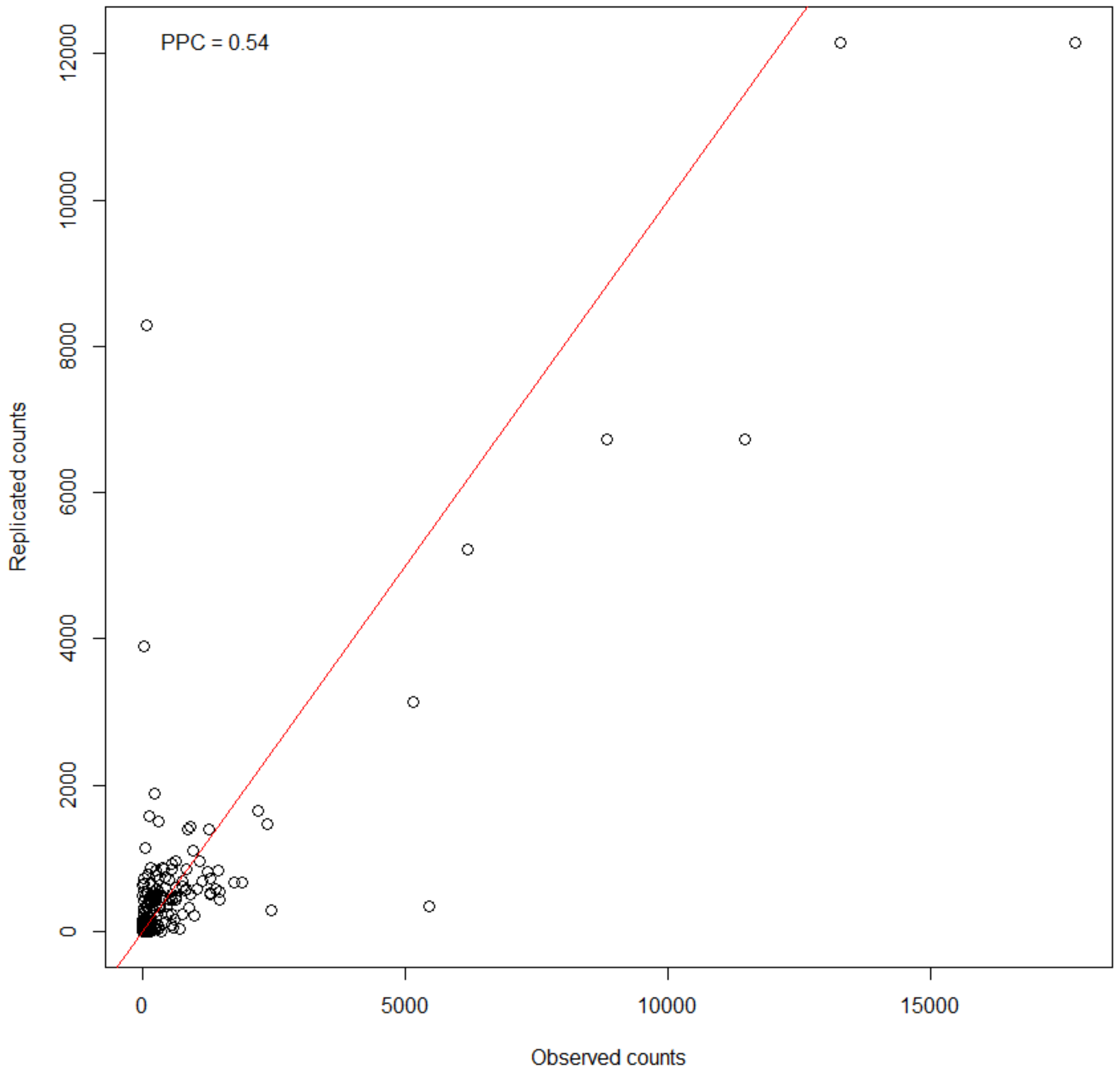

Figure S3-1. Comparison of observed counts and model-replicated counts. The red line indicates the 0,1 relationship. The posterior predictive check (PPC) quantifies the proportion of replicated counts above the observed counts; a PPC = 0.5 is expected for adequate fit.

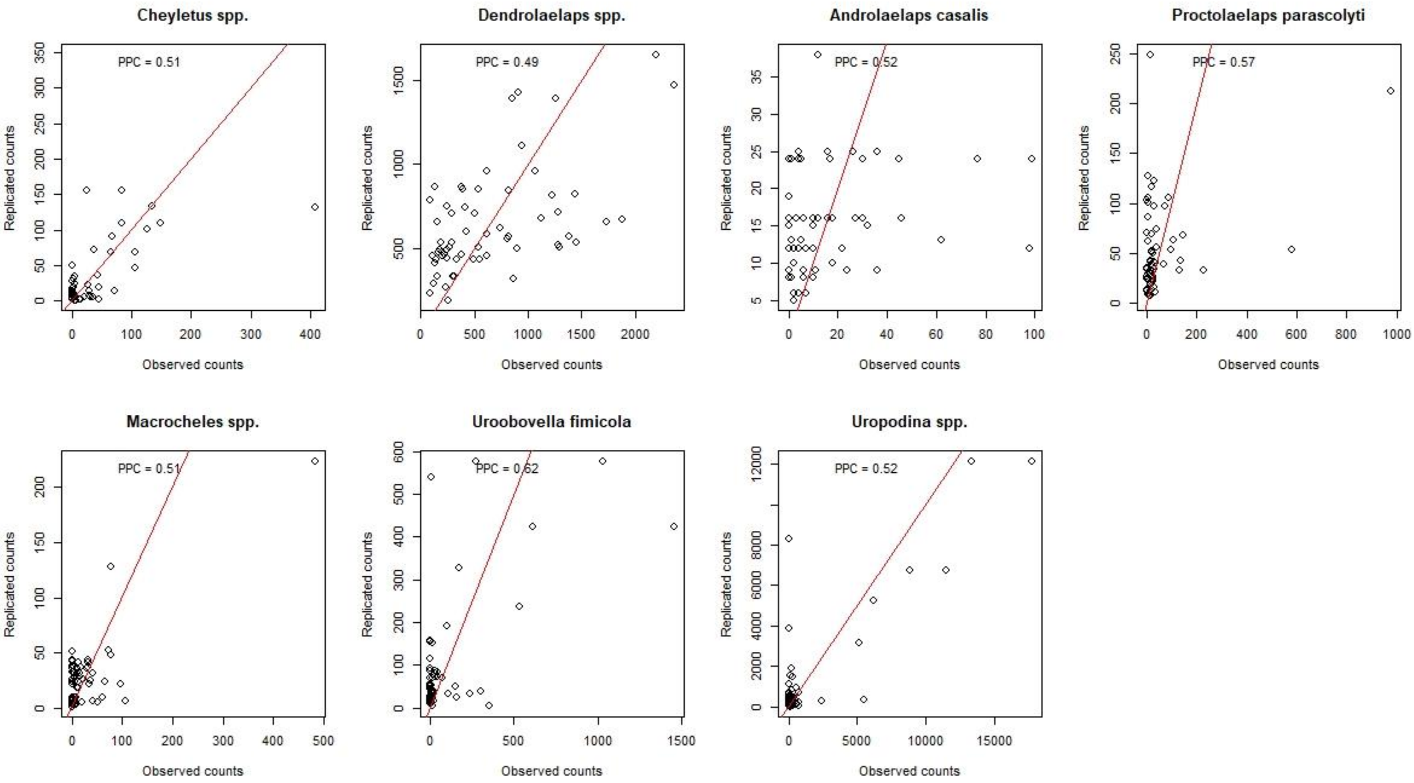

Figure S3-2. Comparison of observed counts and model-replicated counts, per morphospecies. The red line indicates the 0,1 relationship. The posterior predictive check (PPC) quantifies the proportion of replicated counts above the observed counts ; a PPC = 0.5 is expected for adequate fit.

Table S3-1. Rhat values.

**Parameter**	**Morphospecies**	**Modality**	**Rhat**
beta.pred	Cheyletus spp. (CHE)	conventional	0.99961471
beta.pred	Dendrolaelaps spp. (ME1)	conventional	1.00422149
beta.pred	Androlaelaps casalis (ME2)	conventional	1.00085459
beta.pred	Proctolaelaps parascolyti (ME4)	conventional	1.00417468
beta.pred	Macrocheles spp. (ME7)	conventional	1.00066885
beta.pred	Uroobovella fimicola (UR1)	conventional	1.00084038
beta.pred	Uropodina spp. (UR2)	conventional	0.99992939
beta.pred	Cheyletus spp. (CHE)	organic	1.00084247
beta.pred	Dendrolaelaps spp. (ME1)	organic	1.00099342
beta.pred	Androlaelaps casalis (ME2)	organic	1.00151887
beta.pred	Proctolaelaps parascolyti (ME4)	organic	1.00075446
beta.pred	Macrocheles spp. (ME7)	organic	1.00032727
beta.pred	Uroobovella fimicola (UR1)	organic	0.9998926
beta.pred	Uropodina spp. (UR2)	organic	0.99988926
beta.pred	Cheyletus spp. (CHE)	free-range	1.00036749
beta.pred	Dendrolaelaps spp. (ME1)	free-range	0.99991236
beta.pred	Androlaelaps casalis (ME2)	free-range	0.99951034

beta.pred	Proctolaelaps parascolyti (ME4)	free-range	0.99981805
beta.pred	Macrocheles spp. (ME7)	free-range	0.99951844
beta.pred	Uroobovella fimicola (UR1)	free-range	0.99953987
beta.pred	Uropodina spp. (UR2)	free-range	0.99965012
alphadg	Dermanyssus gallinae (DG)		fixed
alphadg	Dermanyssus gallinae (DG)		1.00191013
alphadg	Dermanyssus gallinae (DG)		0.99965291
beta.flock	Cheyletus spp. (CHE)		0.99959922
beta.flock	Dendrolaelaps spp. (ME1)		1.00056249
beta.flock	Androlaelaps casalis (ME2)		1.00039663
beta.flock	Proctolaelaps parascolyti (ME4)		0.99979044
beta.flock	Macrocheles spp. (ME7)		0.99959922
beta.flock	Uroobovella fimicola (UR1)		0.99975344
beta.flock	Uropodina spp. (UR2)		0.99957734
beta.flock.DG	Dermanyssus gallinae (DG)		0.99999338
alpha	Cheyletus spp. (CHE)	conventional	fixed
alpha	Cheyletus spp. (CHE)	organic	0.99986105
alpha	Cheyletus spp. (CHE)	free-range	0.9996903
alpha	Dendrolaelaps spp. (ME1)	conventional	fixed
alpha	Dendrolaelaps spp. (ME1)	organic	0.99961512
alpha	Dendrolaelaps spp. (ME1)	free-range	0.99979896
alpha	Androlaelaps casalis (ME2)	conventional	fixed
alpha	Androlaelaps casalis (ME2)	organic	1.00102493
alpha	Androlaelaps casalis (ME2)	free-range	1.00097036
alpha	Proctolaelaps parascolyti (ME4)	conventional	fixed
alpha	Proctolaelaps parascolyti (ME4)	organic	1.00015619
alpha	Proctolaelaps parascolyti (ME4)	free-range	0.99972301
alpha	Macrocheles spp. (ME7)	conventional	fixed
alpha	Macrocheles spp. (ME7)	organic	1.00000373
alpha	Macrocheles spp. (ME7)	free-range	0.99962006
alpha	Uroobovella fimicola (UR1)	conventional	fixed
alpha	Uroobovella fimicola (UR1)	organic	0.99960734
alpha	Uroobovella fimicola (UR1)	free-range	1.00069861
alpha	Uropodina spp. (UR2)	conventional	fixed
alpha	Uropodina spp. (UR2)	organic	1.00153488
alpha	Uropodina spp. (UR2)	free-range	1.0009188
alphadg	Dermanyssus gallinae (DG)		fixed
alphadg	Dermanyssus gallinae (DG)		1.00191013
alphadg	Dermanyssus gallinae (DG)		0.99965291
beta.seas	Cheyletus spp. (CHE)	AUTUMN	fixed
beta.seas	Dendrolaelaps spp. (ME1)	AUTUMN	fixed
beta.seas	Androlaelaps casalis (ME2)	AUTUMN	fixed
beta.seas	Proctolaelaps parascolyti (ME4)	AUTUMN	fixed
beta.seas	Macrocheles spp. (ME7)	AUTUMN	fixed
beta.seas	Uroobovella fimicola (UR1)	AUTUMN	fixed
beta.seas	Uropodina spp. (UR2)	AUTUMN	fixed

beta.seas	Cheyletus spp. (CHE)	SPRING	1.00058064
beta.seas	Dendrolaelaps spp. (ME1)	SPRING	1.0000975
beta.seas	Androlaelaps casalis (ME2)	SPRING	0.99955887
beta.seas	Proctolaelaps parascolyti (ME4)	SPRING	1.00166962
beta.seas	Macrocheles spp. (ME7)	SPRING	1.00361104
beta.seas	Uroobovella fimicola (UR1)	SPRING	0.99978807
beta.seas	Uropodina spp. (UR2)	SPRING	1.02914811
beta.seas	Cheyletus spp. (CHE)	SUMMER	0.99977783
beta.seas	Dendrolaelaps spp. (ME1)	SUMMER	0.99977864
beta.seas	Androlaelaps casalis (ME2)	SUMMER	0.99951544
beta.seas	Proctolaelaps parascolyti (ME4)	SUMMER	1.00202803
beta.seas	Macrocheles spp. (ME7)	SUMMER	1.0042838
beta.seas	Uroobovella fimicola (UR1)	SUMMER	0.99960857
beta.seas	Uropodina spp. (UR2)	SUMMER	1.02835725
beta.seas	Cheyletus spp. (CHE)	WINTER	0.9995864
beta.seas	Dendrolaelaps spp. (ME1)	WINTER	0.99966079
beta.seas	Androlaelaps casalis (ME2)	WINTER	0.99959972
beta.seas	Proctolaelaps parascolyti (ME4)	WINTER	1.0011229
beta.seas	Macrocheles spp. (ME7)	WINTER	1.00314713
beta.seas	Uroobovella fimicola (UR1)	WINTER	0.99957573
beta.seas	Uropodina spp. (UR2)	WINTER	1.02633448
beta.seas.dg	Dermanyssus gallinae (DG)		fixed
beta.seas.dg	Dermanyssus gallinae (DG)		0.99989192
beta.seas.dg	Dermanyssus gallinae (DG)		0.99961169
beta.seas.dg	Dermanyssus gallinae (DG)		0.9998028
beta.reg	Cheyletus spp. (CHE)	Ain	fixed
beta.reg	Dendrolaelaps spp. (ME1)	Ain	fixed
beta.reg	Androlaelaps casalis (ME2)	Ain	fixed
beta.reg	Proctolaelaps parascolyti (ME4)	Ain	fixed
beta.reg	Macrocheles spp. (ME7)	Ain	fixed
beta.reg	Uroobovella fimicola (UR1)	Ain	fixed
beta.reg	Uropodina spp. (UR2)	Ain	fixed
beta.reg	Cheyletus spp. (CHE)	Drôme	1.00046663
beta.reg	Dendrolaelaps spp. (ME1)	Drôme	0.99988255
beta.reg	Androlaelaps casalis (ME2)	Drôme	1.00080716
beta.reg	Proctolaelaps parascolyti (ME4)	Drôme	0.9995639
beta.reg	Macrocheles spp. (ME7)	Drôme	0.99990598
beta.reg	Uroobovella fimicola (UR1)	Drôme	1.00023259
beta.reg	Uropodina spp. (UR2)	Drôme	1.00120093
beta.reg.dg	Dermanyssus gallinae (DG)		fixed
beta.reg.dg	Dermanyssus gallinae (DG)		1.00081788
mubetapred	Cheyletus spp. (CHE)		1.0000416
mubetapred	Dendrolaelaps spp. (ME1)		1.00157852
mubetapred	Androlaelaps casalis (ME2)		1.00101365
mubetapred	Proctolaelaps parascolyti (ME4)		0.99976735
mubetapred	Macrocheles spp. (ME7)		0.99985868

mubetapred	Uroobovella fimicola (UR1)	1.00000286
mubetapred	Uropodina spp. (UR2)	0.99968712
mubetapred1	all predators	1.0001926

Effect of the remaining covariates

Figures S3-3 to S3-5 provide contrasts from outputs of the Bayesian model according to flock age, region and season respectively.

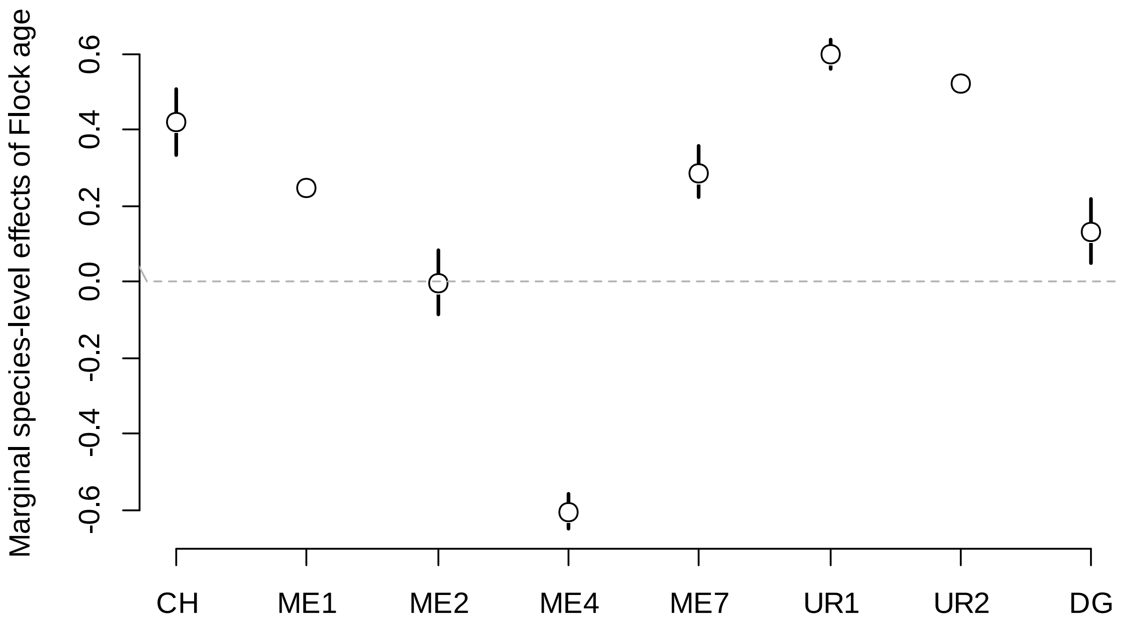

Fig. S3-3. Effect of the flock age.

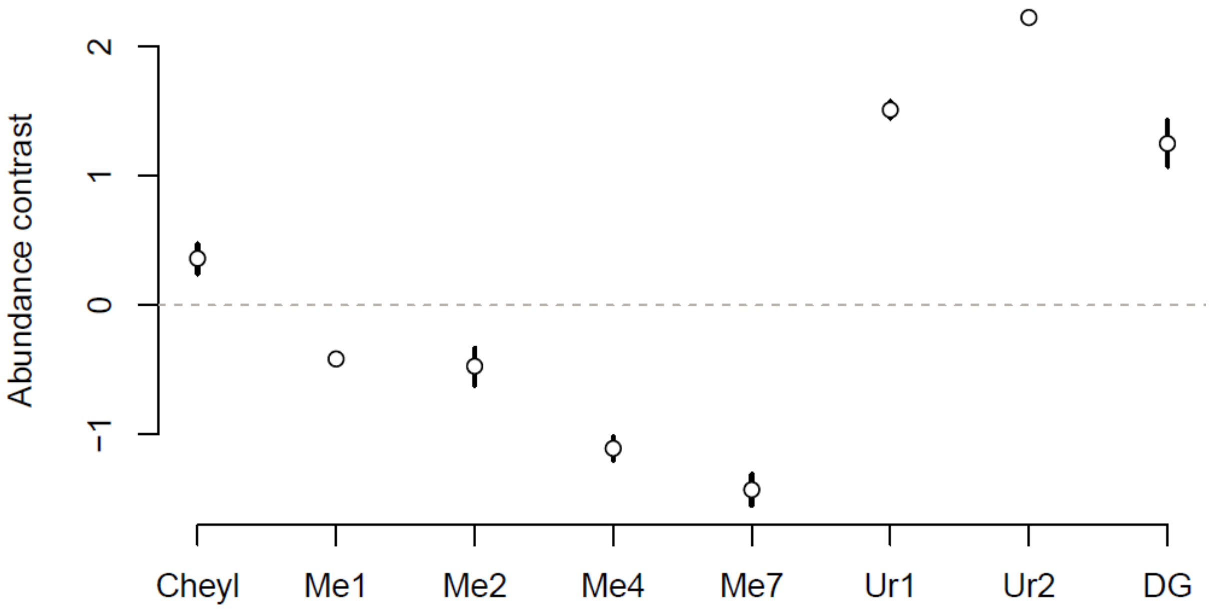

Fig. S3-4. Effect of the region (difference between Ain (reference) and Drôme).

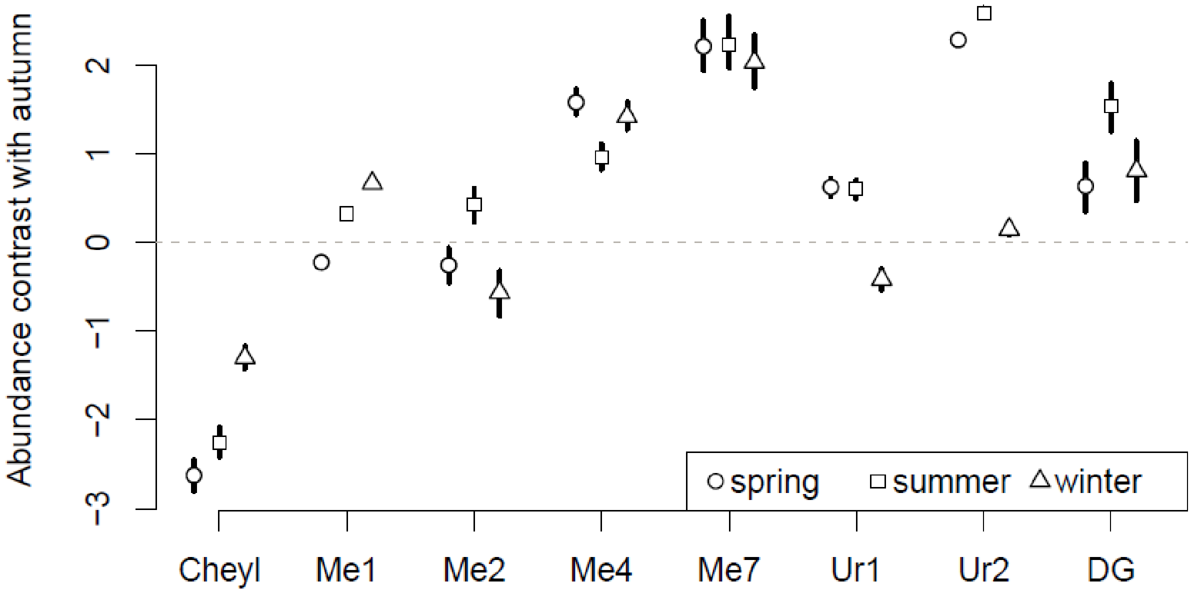

Fig. S3-5. Effect of the season (difference between fall (reference) and the other three seasons).
